# Hollow-Core Fiber-Tip Interferometric High-Temperature Sensor Operating at 1100 °C with High Linearity

**DOI:** 10.3390/mi12030234

**Published:** 2021-02-25

**Authors:** Zhe Zhang, Baijie Xu, Min Zhou, Weijia Bao, Xizhen Xu, Ying Wang, Jun He, Yiping Wang

**Affiliations:** 1 Key Laboratory of Optoelectronic Devices and Systems of Ministry of Education/GuangDong Province, College of Physics and Optoelectronic Engineering, Shenzhen University, Shenzhen 518060, China; 2150190115@email.szu.edu.cn (Z.Z.); 1900453046@email.szu.edu.cn (B.X.); minzhou2020@163.com (M.Z.); wjbao@szu.edu.cn (W.B.); xizhenxu01@163.com (X.X.); yingwang@szu.edu.cn (Y.W.); ypwang@szu.edu.cn (Y.W.); 2 Shenzhen Key Laboratory of Photonic Devices and Sensing Systems for Internet of Things, Guangdong and Hong Kong Joint Research Centre for Optical Fibre Sensors, Shenzhen University, Shenzhen 518060, China; 3 Guangdong Provincial Key Laboratory of Optical Fiber Sensing and Communication, Institute of Photonics Technology, Jinan University, Guangzhou 511443, China; 4 The School of Communication and Information Engineering, Chongqing University of Posts and Telecommunications, Chongqing 400065, China

**Keywords:** high-temperature sensor, hollow-core fiber, annealing-free

## Abstract

Over decades, fiber-optic temperature sensors based on conventional single-mode fibers (SMF) have been demonstrated with either high linearity and stability in a limited temperature region or poor linearity and thermal hysteresis in a high-temperature measurement range. For high-temperature measurements, isothermal annealing is typically necessary for the fiber-optic sensors, aiming at releasing the residual stress, eliminating the thermal hysteresis and, thus, improving the high-temperature measurement linearity and stability. In this article, an annealing-free fiber-optic high-temperature (1100 °C) sensor based on a diaphragm-free hollow-core fiber (HCF) Fabry-Perot interferometer (FPI) is proposed and experimentally demonstrated. The proposed sensor exhibits an excellent thermal stability and linearity (R^2^ > 0.99 in a 100–1100 °C range) without the need for high-temperature annealing. The proposed sensor is extremely simple in preparation, and the annealing-free property can reduce the cost of sensor production significantly, which is promising in mass production and industry applications.

## 1. Introduction

Measurements of high temperatures are of great significance in a variety of industrial applications, such as aerospace [1] and petrochemical engineering [2]. Over the past few decades, various fiber-optic sensors have been developed for high-temperature measurements, such as ultrafast laser-induced fiber Bragg gratings (FBGs) [3,4], sapphire FBG [5], long-period fiber gratings inscribed into a non-photosensitive fiber by a CO_2_ laser [6] or arc discharge [7], fiber Mach-Zehnder interferometers (MZIs) [8,9] and fiber Fabry-Perot interferometers (FPIs) [10,11]. However, fiber-optic high-temperature sensors that are based on the conventional single-mode fiber (SMF) suffer a poor linearity and stability in high-temperature regions before annealing treatments [12,13]. This can be attributed to the residual stress releasing and different thermo-expansion coefficients of the fiber cladding (silica) and Ge-doped core when the fiber is heated to the softening point temperature of the material [14,15]. To eliminate the residual stress and improve the sensor’s linearity and stability, the isothermal annealing treatment is necessary and effective [16]. Moreover, to avoid the introduction of extra stress during the annealing process, the speed of heating and cooling is strictly limited due to the different thermo-expansion coefficients of the fiber core and cladding.

Alternatively, the naissance of the hollow-core fiber (HCF), which is constitutive of pure silica without any ion doping [17], brings about a new base for fiber-optic high-temperature sensors. For instance, in 2011, M. Ferreira proposed an HCF-based diaphragm-free FPI that can work in 1000 °C [18]. However, the unbefitting HCF make the FPI exhibit a poor fringe visibility (~2 dB), as well as mode interference noise.

In this article, an annealing-free high-temperature (1100 °C) sensor based on diaphragm-free HCF FPI is proposed and experimentally demonstrated. A 180-µm-long HCF with an air core diameter of 4 µm, which is experimentally demonstrated as the best parameter, is fusion-spliced with a lead-in SMF, forming a high-quality diaphragm-free FPI with fringe visibility higher than 10 dB. High-temperature cycle tests ranging from 100 to 1100 °C indicate a high response linearity (>0.99) and stability without the need for isothermal annealing treatments. The proposed sensor is simple in preparation, and the annealing-free property of the sensor will reduce the cost significantly, making itself a promising candidate for industrial production and applications.

## 2. Sensor Fabrication and Working Principle

**Figure 1:**
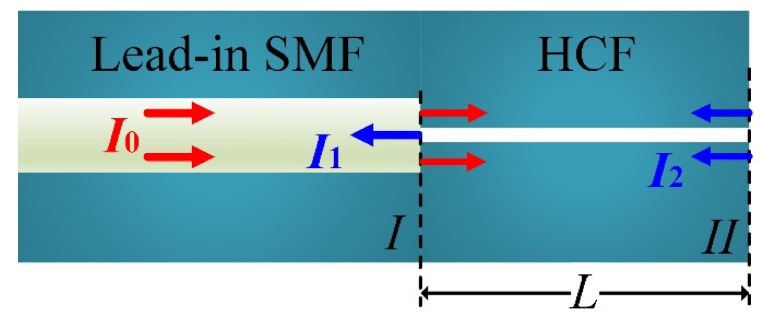
Schematic illustration of the proposed FPI sensor. FPI: Fabry-Perot interferometer, SMF: single-mode fiber, *I*_1_/*I*_2_: intensity ratio, *I*_0_: the intensity of the incident light, HCF: hollow-core fiber *L*: length of HCF, interface I: the SMF end facet, interface II: the HCF end facet.

**Figure 2:**
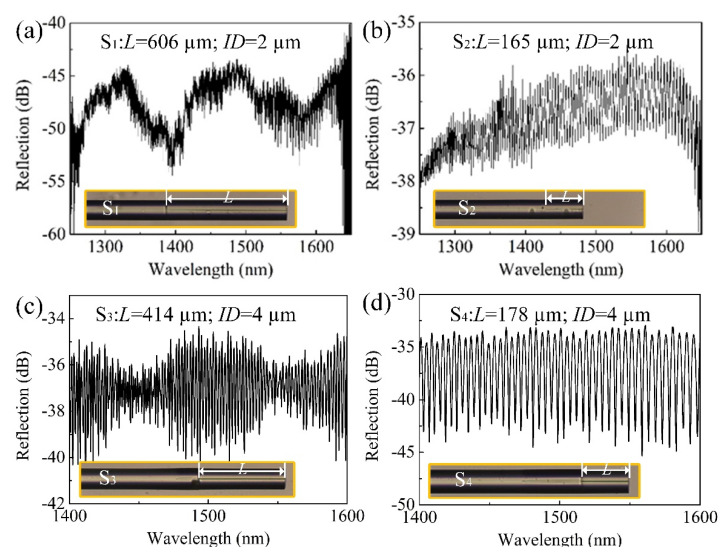
Reflection spectra of the four prepared FPIs with different HCF parameters: (**a**) inner diameter (*ID*): 2 µm and *L*: 606 µm, (**b**) *ID*: 2 µm, and *L*: 165 µm, (**c**) *ID*: 4 µm and *L*: 414 µm and (**d**) *ID*: 4 µm and *L*: 178 µm.

The proposed fiber-optic FPI sensor, as illustrated in Figure 1, is prepared by fusion splicing a length of HCF (INNOSEP-TSP07515, CN) with a lead-in SMF (Corning “SMF-28e”, Wilmington, NC, USA), followed by cutting off the HCF with the assistance of a homemade microscope to precisely control the remaining length. The microscope of the cross-section of the HCF is available in our previous work [19]. As shown in Figure 1, light propagates in the lead-in SMF are partially reflected at the end face of SMF; it is worth noting that part of the core light was transmitted into the silica cladding of the HCF, which hardly suffers from Fresnel reflection due to the small refractive index (RI) difference between the SMF core and the silica cladding of the HCF. To preciously express the reflected intensity, we defined the ratio *γ* parameter that characterized the proportion of light that transmitted into the air core of the HCF, i.e., suffering Fresnel reflection at a conventional SMF end facet. As such, the reflection intensity can be expressed as

(1) $$I_{1}=I_{0}\gamma R_{1},$$

where *I*_0_ is the intensity of the incident light, *γ* is the intensity ratio of the light that is transmitted into the air core of the HCF and *R*_1_ is the reflectivity of the SMF end facet, i.e., “interface I”. A fraction of the light transmitted in the lead-in SMF is coupled into the silica cladding of the HCF due to the smaller core of HCF than the lead-in SMF. After transmitting for length *L*, i.e., the length of HCF in the HCF cladding, light is reflected at the HCF end facet, i.e., “interface II”, transmitting back and recoupling into the lead-in SMF. The reflection intensity can be expressed as

(2) $$I_{2}=I_{0}(1-\gamma )R_{2},$$

where *R*_2_ is the reflectivity of the HCF end face. For simplify the purpose, the optical transmission loss in the HCF cladding is neglected, given that the HCF is short. Here, the intensity ratio of the light that transmitted into the air core of the HCF, i.e., *γ*, is determined by the air core diameter of the HCF. To be specific, the mode field of the lead-in SMF is Gaussian-distributed with a diameter of ~10 µm. A larger air core renders a larger *γ*, as shown in Figure 1. As such, the fringe visibility of the FPI, which is determined by the intensity ratio (*I*_1_/*I*_2_) of the two reflection beams, can be altered flexibly by optimizing the inner diameter (*ID*) of the HCF according to Equations (1) and (2). However, when the *ID* of the HCF is larger than the mode field diameter of the SMF, a little fraction of light can propagate into the silica cladding of the HCF, i.e., *γ* ≈ 1, resulting in a poor fringe visibility (~2 dB), as reported in reference [18]. The poor fringe visibility of the FPI spectrum imposes difficulties for peak tracking and wavelength demodulations. Figure 2 shows the reflection spectra of four prepared FPI sensor samples (S_1_–S_4_) with HCF *ID*s of 2 µm (S_1_ and S_2_) and 4 µm (S_3_ and S_4_), respectively. The HCF with an *ID* of 4 µm is considered as the best choice for the preparation of the FPI sensors with higher fringe visibility.

**Figure 3:**
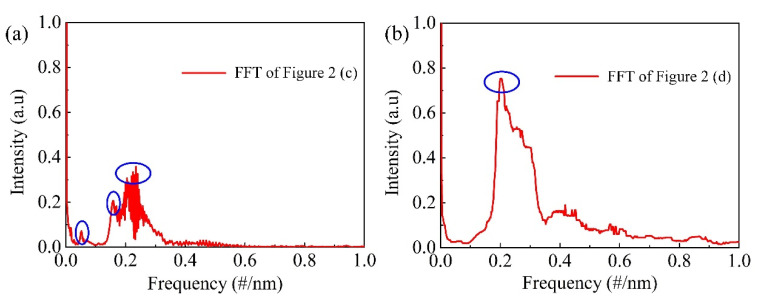
Frequency spectra of the interference spectra of (**a**) S_3_ and (**b**) S_4_ by Fast Fourier-Transform (FFT).

In addition to the *ID*, the length *L* of the HCF is another important parameter that affects the spectra quality of the FPI. To be specific, multiple modes will be excited when the HCF is long enough, resulting in a superimposed interference spectrum. As can be clearly seen in Figure 2c, where the length *L* of the HCF is ~414 µm, the interference spectrum is characterized by dense fringes modulated by a large envelope, implying a multibeam interference. Figure 3a shows the “spectrum of spectrum” result by applying Fast Fourier-Transform (FFT) to the interference spectrum shown in Figure 2c; the multiple frequency components can be clearly identified from Figure 3a. As such, a proper HCF length is vital for the spectrum quality. By shorting the HCF (~178 µm), a typical two-beam interference spectrum can be obtained, as shown in Figure 2d, and the FFT result, which is shown in Figure 3b. Similar results can be found in Figure 2a,b, where the *ID* of the employed HCF is 2 µm.

## 3. High-Temperature Properties of the Proposed Sensor

**Figure 4:**
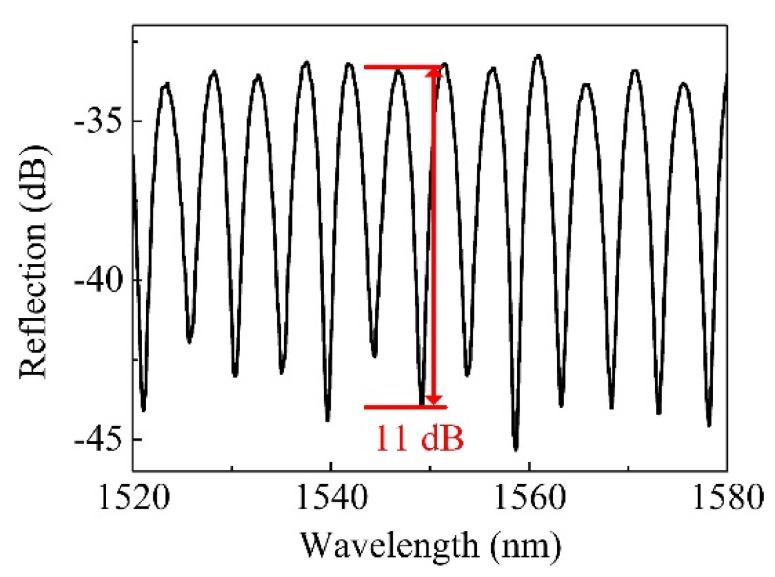
Reflection spectrum of a prepared FPI sensor with optimized HCF parameters (*ID*: 4 µm and *L*: 180 µm).

**Figure 5:**
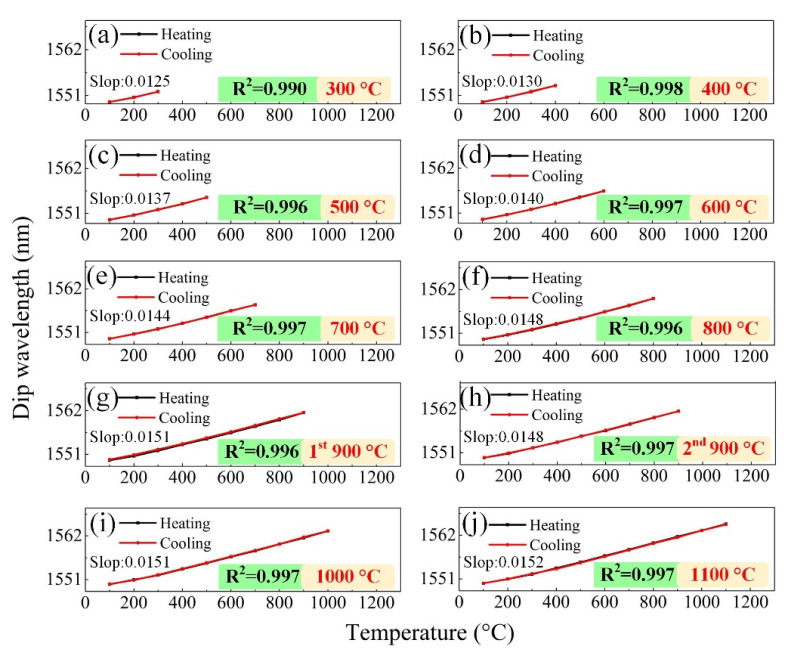
Wavelength of the tracked dip versus temperature for ten temperature ranges of (**a**) 100–300 °C, (**b**) 100–400 °C, (**c**) 100–500 °C, (**d**) 100–600 °C, (**e**) 100–700 °C, (**f**) 100–800 °C, (**g**) 1st 100–900 °C (**h**) 2nd 100–900 °C, (**i**) 100–1000 °C, and (**j**) 100–1100 °C.

After the parameters (*ID* and *L* of HCF) optimization, a high-quality FPI sensor was prepared, and the reflection spectrum is shown in Figure 4, where the fringe visibility is ~11 dB at ~1550 nm. The length of the HCF, i.e., cavity length *L* of the FPI, is ~180 µm. The high-temperature sensing characteristics of the fiber-tip HCF FPI was then experimentally studied by placing the sensor into a high-temperature furnace (CarboliteGero, GHA 12/750, Hope, UK) that can reach 1200 °C with an accuracy of ±1 °C. It is worth noting that the temperature sensitivity is also wavelength-dependent; this relationship was detailed in our previous work [19]. For simplicity and clarifying purposes, a wavelength of the interference dip near 1550 nm was employed as the temperature indicator. The temperature was stepwise raised and then cooled passively. Each temperature point was held for ~10 min to ensure a stable temperature distribution in the oven before recording. The wavelength of the tracked dip in the interference spectrum presented a “red shift”, with the temperature increasing from 100 to 300 °C. The observed “red shift” can be attributed to a combined impact of the thermo-optic and thermal expansion effects of the silica, where the thermal expansion contributes little compared to the thermo-optic effect. The temperature sensitivity can be expressed as

(3) $$\frac{d\lambda }{dT}=\lambda [\frac{1}{n_{si}^{eff}(T)}\frac{dn_{si}^{eff}(T)}{dT}+\alpha ]$$

where $dn_{si}^{eff}(T)/dT$ and α are the thermo-optic and thermal expansion coefficients of silica, respectively. The $n_{si}^{eff}\left(T\right)$ increases with the temperature; as such, the contribution of the thermo-expansion effect cannot be neglected at high temperature conditions, according to Equation (3). A detailed analysis was presented in our previous work [19]. During the cooling process, no thermal hysteresis and wavelength separation from the heating process were observed, as shown in Figure 5a. To explore the high-temperature response properties of the sensor, the maximum oven temperature was increased from 400 to 800 °C in steps of 100 °C, the results of which are clearly shown in Figure 5b–f, where the thermal hysteresis or wavelength separation between heating and cooling were not observed and the measurement linearity at each temperature range were all higher than 0.99. A little wavelength separation of ~0.2 nm was observed between the heating and cooling processes when the temperature increased to 900 °C, as depicted in Figure 5g. We believe that the small wavelength separation may be attributed to stress releasing, where the stress was introduced by a little squeezing of the HCF and led-in SMF during the arc discharge thermal fusion. Despite the small wavelength separation (~0.2 nm), the measurement linearity was still higher than 0.99. A repeated high-temperature test from 100 to 900 °C was performed; after which, the wavelength separation was eliminated, and an improved linearity of 0.997 was convinced, as shown in Figure 5h. Furthermore, temperature experiments in a temperature range of 100–1000 °C and 100–1100 °C were performed, respectively, where no thermal hysteresis and high linearity (>0.99) were demonstrated experimentally. The results are shown in Figure 5i,j, respectively.

During the whole high-temperature measurements, the tracked dip exhibited a linear “red shift” with the temperature increasing, indicating a linear RI increase of the HCF cladding. The cooling curve coincided well with the heating curve, and no thermal hysteresis was observed within a temperature range from 100 to 1100 °C. The observed high-temperature response of the proposed FPI sensor agreed well with our hypothesis that the employment of pure-silica HCF as the high-temperature sensing element could dismiss the complicated isothermal annealing processes. The temperature sensitivity at each temperature test range was 12.5, 13.0, 13.7, 14.0, 14.4, 14.8, 15.1, 14.8, 15.1 and 15.2 pm/°C, respectively. The achievable minima temperature change was determined by the wavelength resolution of the OSA (OSA; optical spectrum analyzer) or interrogator. In our experiments, the employed OSA had a wavelength resolution of 0.02 nm. Considering a temperature sensitivity of 15 pm/°C, the achievable measured minima temperature change was ~1.3 °C.

## 4. Conclusions

A high-linearity (>0.99) annealing-free fiber-optic high-temperature (1100 °C) sensor was experimentally demonstrated. The proposed sensor was diaphragm-free and simply prepared by fusion splicing. The impact of the HCF length *L* and *ID* on the interference spectrum qualities were experimentally studied. Benefitting from pure-silica HCF, a high measurement linearity (>0.99) of the sensor was demonstrated in a temperature ranging from 100 to 1100 °C. The proposed sensor dismissed the complicated isothermal annealing process effectively. Such a fiber-optic high-temperature sensor can reduce the cost of sensor preparation significantly and may find vital applications in the industry, such as aero engines and melting furnaces.

## Data Availability

The datasets are available from the corresponding author on reasonable request.
